# Urinary neprilysin in the critically ill patient

**DOI:** 10.1186/s12882-017-0587-5

**Published:** 2017-05-25

**Authors:** Sahra Pajenda, Karl Mechtler, Ludwig Wagner

**Affiliations:** 1 Department of Internal Medicine III, Division of Nephrology and Dialysis, Medical University of Vienna, Währinger Gürtel 18-20, 1090 Vienna, Austria; 2 ProtChem Facility, IMP–IMBA - Research Institute of Molecular Pathology, Dr. Bohr Gasse 3, 1030 Vienna, Austria

**Keywords:** Acute renal injury, Biomarkers, Proximal tubular injury

## Abstract

**Background:**

Critically ill patients in intensive care face hazardous conditions. Among these, acute kidney injury (AKI) is frequently seen as a result of sepsis. Early diagnosis of kidney injury is of the utmost importance in the guidance of interventions or avoidance of treatment-induced kidney injury. On these grounds, we searched for markers that could indicate proximal tubular cell injury.

**Methods:**

Urine samples of 90 patients admitted to the intensive or intermediate care unit were collected over 2 to 5 days. The biomarker neprilysin (NEP) was investigated in urine using several methods such as dot blot, ELISA and immunofluorescence of urinary casts. Fifty-five healthy donors acted as controls.

**Results:**

NEP was highly significantly elevated in the urine of patients who suffered AKI according to the KDIGO criteria in comparison to healthy controls. It was also found to be elevated in ICU patients without overt signs of AKI according to serum creatinine changes, however they were suffering from potential nephrotoxic insults. According to our findings, urinary NEP is indicative of epithelial cell alterations at the proximal tubule. This was elaborated in ICU patients when ghost fragments and NEP^+^ microvesicles were observed in urinary sediment cytopreparations. Furthermore, NEP^+^ immunofluorescence of healthy kidney tissue showed staining at the proximal tubules.

**Conclusions:**

NEP, a potential marker for proximal tubular epithelia, can be measured in urine. This does not originate from leakage of elevated serum levels, but indicates proximal tubular cell alterations such as brush border severing, which can heal in most cases.

## Background

Acute kidney injury (AKI) is a common complication of serious infections such as sepsis. About 20–50% of patients suffering from the disease suffer AKI in various stages depending on the severity of the septic symptoms [1, 2]. In a minor group of these patients it may lead to end stage kidney disease with the necessity of renal replacement therapy (RRT). However, in most cases patients regain kidney function when antimicrobial treatment is initiated and hemodynamic parameters are stabilized rapidly.

The predominant targets of cellular injury are proximal epithelial cells, although glomerular lesions have also been described [3]. Loss of brush border and disintegration of tubular cells have been demonstrated [4]. The clinical observation that a phase of oliguria is followed by a polyuric episode [5] preceding recovery of kidney function is reflective of a process of cellular repair mechanisms, especially at the tubular compartment [6–8].

Major kidney insults result in changes of fractional excretion of sodium (FeNa), a decrease in urine output and finally a rise in serum creatinine (sCr). These parameters are still the most widely used diagnostic indicators of acute kidney injury [9]. Nevertheless, laboratory findings such as serum creatinine indicate kidney damage due to an injury that occurred 12–24 h earlier. It represents loss of more than half of the glomerular filtration rate (GFR) [10]. The lack of diagnostic indicators for early phases of tubular cell injury, ideally detectable in urine, represents a challenge for biomarker development. A huge array of urine proteomic data has been provided in the past years [11–15]. KIM-1 [16], NGAL [17] and a MALDI MS-based platform was tested for early detection for AKI [15]. Furthermore IGFBP7 [18] and TIMP-2 [19] are simply examples which investigators had been testing for their specificity and clinical value in detecting the extent of kidney injuries. However, some of them are associated with drawbacks. The NephroCheck measuring TIMP2 and IGFBP7 has been developed very recently as a rapid bedside diagnostic tool. This yielded promising results in validation studies [19, 20].

Inspired by the rapid pace of research and the development of specific inhibitors [21, 22], we selected neprilysin, also called neutral endopeptidase (NEP) representing a single-pass membrane glycoprotein with zinc-dependent endopeptidase activity and a short cytosolic tail [23], as a promising diagnostic marker. The expression in the brush border of proximal tubular cells, the most vulnerable stretch of epithelial cells, was one of the main motives to start testing its occurrence in urine [24]. In addition this protein was found among many others in urinary exosomes [25].

Furthermore, its involvement in physiological processes such as blood pressure regulation and the development of inhibitors has brought this protein to the forefront of medical interest [26, 27]. As a marker for kidney function that is measured in urine in patients undergoing cardiac surgery it has been shown to significantly increase 3–36 h post-surgical intervention when tubular damage was thought to occur. It was thus hypothesized that NEP constitutes an indicator of early tubular injury. However, the study had been performed in the specific clinical setting of cardiac surgery [28].

Following this line of thought, we collected urine from critically ill and sepsis patients over a time period of 2–5 days in order to evaluate whether this diagnostic marker can be detected in urine under these conditions. In addition, urinary sediment has been investigated using immunofluorescence for NEP positive tubular cells and cellular fragments.

## Methods

### Study population

Critically ill patients (*n* = 90) fulfilling sepsis criteria [29] at the Intensive Care Unit (ICU) Department of Medicine III and the Department of Nephrology, Medical University of Vienna have been continuously enrolled in this study between 2009 and 2013. Informed consent was obtained from the participants. Inclusion criteria were defined as age above 18 years and admission to intensive or intermediate care of the Department of Medicine III. End Stage Renal Disease (ESRD) with the need for renal replacement therapy constituted an exclusion criterion, whereas patients who had had kidney transplantation with or without delayed graft function were included (*n* = 6). Participation in another pharmacotherapeutic study requiring blood or other sampling was an exclusion criterion. The date and time of study enrollment was defined as 1, with the first sample being taken at this point in time.

Plasma aliquots of 21 patients enrolled between November 2011 and November 2012 were kept frozen at −80 °C on account of the continuous research project regarding AKI-specific biomarkers. Plasma samples of these patients were thawed and analyzed with the sole aim of answering the question of whether urine NEP originates from leakage via the glomerular barrier or from tubular compartments. A time-matched plasma/urine ratio was established for these patients, however, the main goal of this study was to investigate urinary neprilysin in critically ill patients.

Patients’ clinical data, demographics, medical history, medication and laboratory data were recorded from hospital files and databases of the general hospital of Vienna. Patients were subdivided into two groups (AKI vs. No AKI) and AKI was graded according to the KDIGO guidelines [30]. Fifty-five healthy donors served as controls. Their urine was obtained without the presence of a catheter. The study was approved by the local Ethics Committee of the Medical University of Vienna.

### Sample collection

All patients had an indwelling Foley catheter and urine specimens were collected from the chamber which represented fluid no older than 2 h. Samples were obtained at an interval of 24 h over 2 to 5 consecutive days, with the exception of the second sample which was obtained 8–12 h following the first sample. Serum creatinine was evaluated at exactly the same time point when NEP was measured. Within 1 h of collection, samples were centrifuged at 3000 RPM and the resultant supernatant was immediately stored frozen at −80 °C for further analysis in several aliquots.

### Selecting proteins to be analysed

Initial analyses comprised the gene product of C20orf3 (chromosome 20 open reading frame 3), C6orf62 (chromosome 6 open reading frame 62) and CD133 (prominin). NEP could be verified by searching the human protein atlas for proteins expressed in the proximal kidney tubule [31]. Similarly it has been previously demonstrated that NEP antibodies could specifically select proximal epithelial tubular cells (PTC) [32].

### Cytospin preparation

For cytospin preparations, the urinary sediment was re-suspended in tissue culture medium RPMI 1640 containing 10% fetal calf serum (GIBCO, Grand Island, N.Y). 200 μl of the resultant cell suspension was applied to slides through a funnel of a cyto-centrifuge (Cytospin 3 Shandon, England). Following this, samples were loaded and spun at 1200 RPM for 4 min. Cytoslides were air-dried and either wrapped in aluminum foil and stored at −20 °C or used immediately for immunofluorescence staining.

### Immunofluorescence

Immunofluorescence staining was performed by fixing the slides in acetone for 5 min.

The primary antibody (NCL-L-CD10–270) diluted 1:300 in phosphate-buffered saline (PBS) and blocking solution (BSA), was added to each sample. After incubation in a moist chamber at 4 °C overnight the slides were washed twice with PBS. The secondary antibody (Alexa fluor 488, goat anti-mouse, Invitrogen), diluted 1:300 in PBS and BSA blocking solution, was applied and incubated for 2 h at 4 °C. Then 40 μl of DAPI was applied for the last 5 min of incubation followed by washing with Tween PBS (TPBS) and subsequently PBS for 10 min each. As a final step, slides were mounted in Vectashield mounting medium (Vector Laboratories), and investigated by confocal microscopy (Zeiss Axiovert). Images were captured via Zen 12 software and further edited using Adobe Photoshop CS6.

Kidney tissue staining was similarly performed as described above for cytopreparations.

### Immunoblotting

For investigative purposes, we established a NEP dot blotting test as described in brief: 35 μl of urine was applied to individual wells of a dot blotting device (Schleicher & Schüller, Minifold I). The fluid was filtered through the inserted nitrocellulose membrane by applying vacuum to the bottom chamber. The resultant blot was then immersed in PBS after 10 min of air drying and blocked in one blocking solution (KPL, Gaitersburg, MD, USA) for 30 min. The blocked membrane was then incubated in 10 ml of anti-NEP antibody (NCL-L-CD10–270) overnight at 4 °C followed by the second incubation with the detection antibody (HRP-conjugated goat anti-mouse, Dako, P0447). Each incubation period was followed by a 10-min TPBS wash twice. Finally, the site of antibody binding was visualized using the chemiluminescence reagent (BM Chemiluminescence Blotting Substrate POD, Roche) and developed under the imager system Lumi-Imager F1 (Roche) using Fusion FX (Vilber Lourmat) software for recording pictures and evaluating dot intensity.

### ELISA

Quantitative measurements of urine NEP of 90 patients with sepsis and 55 controls were taken with a 96-well plate human NEP ELISA (RayBiotech, Inc.) as described in the company’s ELISA Kit protocol. The standard dilution series as well as urine samples were applied to the wells and incubated at 4 °C overnight. The next morning, the plate was washed three times with the washing buffer provided using an automated ELISA plate washing machine (ELx50 Auto Strip Washer, BIO-TEK INSTRUMENTS, INC). A detection antibody for human NEP was added to each well and incubated for 1 h at room temperature with constant shaking. After a washing step, HRP-streptavidin was incubated for 45 min at room temperature. Following the final washing step, the TMB substrate/chromogen solution was added for developing (15 min of incubation at room temperature under light protection). Subsequently, the stop solution was pipetted into each well and the plate was read at 450 nm by the ELISA reader (Synergy H1 Hybrid Reader, BioTek).

### Statistical analysis

NEP data are presented, using scatter dot plots, wherein the middle line represents the median. Different groups were compared with the Mann Whitney test using GraphPad Prism5 software. A p-level of <0.05 was considered statistically significant. Sensitivity, specificity and ROC curve analyses, correlation of plasma and urine NEP according to Pearson were all calculated using the GraphPad Prism.

## Results

### Protein analysis

**Fig. 1:**
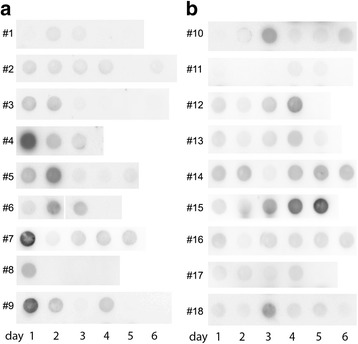
NEP *Dot blot* analyses of 18 patients. **a** *Dot blot* of 9 patients over 3–6 days of follow-up, presenting with elevated levels of NEP in the *dot blot* method within the first 24 h of admission to ICU. **b** *Dot blot* analyses for course of NEP secretion of other 9 patients in follow-up, presenting an increase during ICU stay

In an initial experiment, we observed during the analysis of urinary NEP levels with dot blot measurements that this protein might be an early indicator of kidney injury. In order to follow up on this, we performed dot blot measurements on 18 arbitrarily chosen patients **(**Fig. 1a **)**. In half of the patients, NEP already showed its peak levels 24 h before serum creatinine (sCr) rise. However, this was not the case in all of the patients as shown in Fig. 1b.

**Fig. 2:**
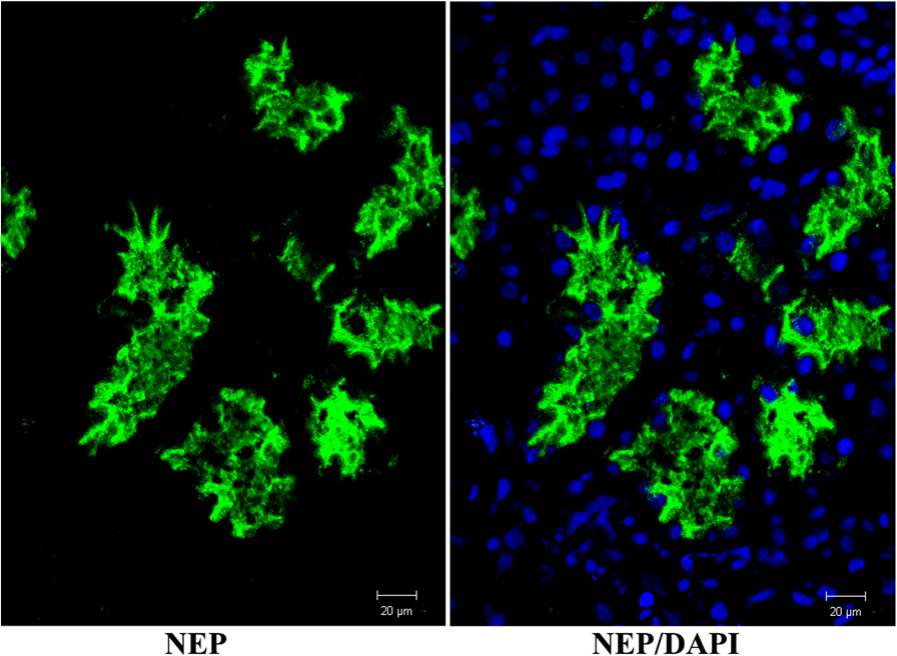
NEP staining of normal healthy kidney tissue obtained from tumor nephrectomy. An area showing proximal kidney tubules with prominent apical staining of epithelial cells is depicted. Magnification indicated at the *bottom* of each picture

We performed immunofluorescence staining on healthy kidney tissue obtained from tumor nephrectomies using the same immunoreagents. This staining confirmed NEP expression in proximal tubules in all four samples (Fig. 2).

**Fig. 3:**
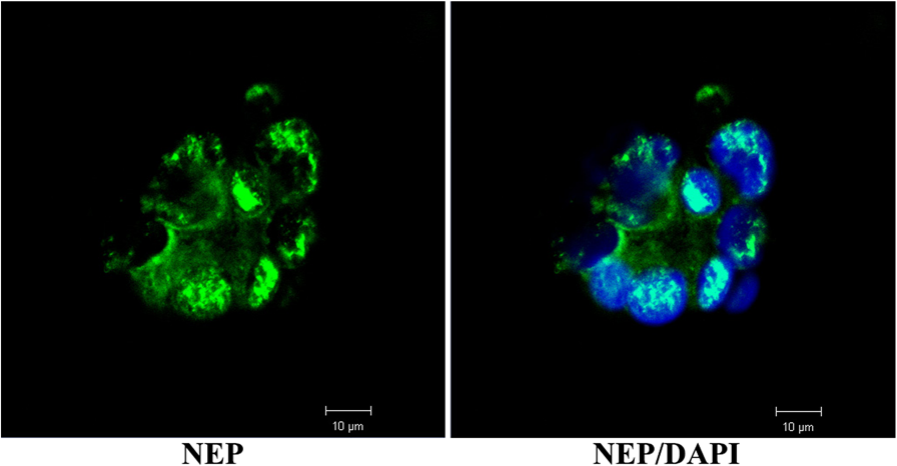
NEP staining of urinary cells. Urinary cells from a patient having undergone renal transplantation 5 days beforehand. Proximal tubular cell ghosts with ruptured cell membranes and spreading DNA due to nuclear membrane damage are depicted. Magnification indicated at the *bottom* of each picture

As a second step, cytospins had been prepared from urinary cells and casts obtained from 8 arbitrarily chosen sepsis patients, which were similarly stained for NEP expression. Interestingly, NEP-positive tubular epithelial cells were rarely detected. In contrast, small microvesicles were demonstrated in 5 out of 8 cases. In addition, urinary sediment from kidney allograft recipients was taken to evaluate proximal tubular cells in accordance with this method using confocal microscopy. As depicted in Fig. 3, cell ghosts and fragmented proximal tubular cells were visualized.

### Quantitative measurement of urinary NEP by ELISA

A commercially available ELISA test was employed for the quantitative measurement of NEP.

Ninety patients (32 female and 58 male), of whom 24 (26.67%) had undergone cardiopulmonary resuscitation, were included and tracked for at least 48 h (Table 1). The mean age of patients was 60 ± 16 years. Sixty-five (72.22%) of them were diagnosed with pneumonia as the predominant focus of infection. Urinary tract infection was verified as the source of infection in 11 patients (12.22%). In 17 patients (18.89%), sepsis origin varied from spontaneous bacterial peritonitis (SBP), septic shock and wound infections to gastrointestinal infections.

**Table 1:** Demographics of the study cohort. Demographic table of the study cohort showing patients characteristics including age, sex, sepsis origin, sedoanalgesia, intubation, need for catecholamines and stage of kidney injury according to the KDIGO criteria

	No AKI	Stage 1	Stage 2	Stage 3	Controls	Total
Number of patients	22	42	12	14	55	90
	24.44%	46.67%	13.33%	15.56%		
Age, mean ± SD	60 ± 16	60 ± 16	59 ± 17	60 ± 16		60 ± 16
Female / Male	9 / 13	17 / 25	4 / 8	2 / 12	40 / 15	32 / 58
Sepsis originated pneumonia	19	30	9	7		65
	86.36%	71.43%	75.00%	50.00%		72.22%
Sepsis originated urinary tract	0	5	1	5		11
	0.00%	11.90%	8.33%	35.71%		12.22%
Sepsis origin others	3	8	2	4		17
	13.64%	19.05%	16.67%	28.57%		18.89%
CPR	7	12	4	1		24
	31.82%	28.57%	33.33%	7.14%		26.67%
Sedoanalgesia	17	29	7	7		60
	77.27%	69.05%	58.33%	50.00%		66.67%
Intubation	18	28	7	6		59
	81.82%	66.67%	58.33%	42.86%		65.56%
Catecholamines	15	23	7	7		52
	68.18%	54.76%	58.33%	50.00%		57.78%
NEP positive	9	32	10	9	5	60
NEP positive %	40.91%	76.19%	83.33%	64.29%	9.09%	66.67%
NEP negative	13	10	2	5	50	30
NEP negative %	59.09%	23.81%	16.67%	35.71%	90.91%	33.33%

**Fig. 4:**
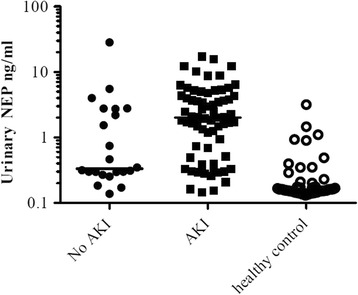
*Column scatter graph* comparing NEP in patients without AKI, with AKI and healthy control. The *scatter plot* of NEP (highest ELISA values detected during the observation period) of 90 sepsis patients is depicted, and the patients were divided into 2 groups: no AKI including patients with a history of CKD (*n* = 22), patients with AKI (*n* = 68) dynamics reaching one of the defined KDIGO stages and healthy individuals (*n* = 55)

**Fig. 5:**
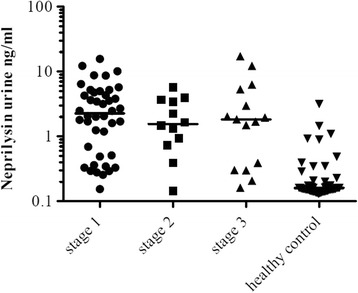
*Column scatter graph* of patients with AKI and healthy control. *Scatter plot* analysis of patients suffering AKI, divided into stage 1 (*n* = 42), stage 2 (*n* = 12) and stage 3 (*n* = 14) by KDIGO in comparison to healthy controls. The highest level detected during the observation period is depicted

Based on serum creatinine, patients were subdivided into 2 categories: patients without the presence of AKI including patients with a history of chronic kidney disease CKD (*n* = 22) and patients with AKI (*n* = 68), shown in Fig. 4. Patients suffering AKI had been further subdivided and graded according to the KDIGO criteria (stage 1, *n* = 42; stage 2, *n* = 12; stage 3, *n* = 14), depicted in Fig. 5. This was established by medical history data. As a control, healthy urine donors were chosen (*n* = 55). Surprisingly, five healthy male donors presented elevated NEP values which had not been described before. By further investigating the human protein atlas, it proved to be clear that the prostate epithelium exhibits high expression of NEP. Despite this fact, male and female control donors were included for statistical analyses. In this study all ICU and intermediate care patients had an indwelling urinary catheter, whereas control donors delivered spot urine.

Of the 90 critically ill patients enrolled in this study, 68 patients had an acute kidney injury and 22 patients showed stable kidney function (75.56% versus 24.44%).

Using a cut-off value of 0.5 ng/ml for NEP, sensitivity of this urinary test was 75.00% (CI 95%, 0.63–0.84), with a specificity of 59.09% (CI 95%, 0.36–0.79). A positive predictive value was calculated at 85.00% (CI 95%, 0.73–0.93) and a negative predictive value at 43.33% (CI 95%, 0.25–0.62).

**Fig. 6:**
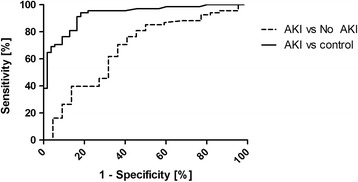
ROC curve of maximum NEP in critically ill AKI patients versus healthy controls (*solid line*). ROC curve of maximum NEP in critically ill AKI patients versus no-AKI patients (*hatched line*). Receiver-operating characteristics curve (ROC)

As demonstrated in Fig. 4, there was highly elevated NEP in patients suffering AKI at various AKI stages in comparison to the healthy control. In the performed ROC curve analysis shown in Fig. 6 the AUC value was 0.93 (95% CI 0.88–0.97, SE = 0.02, *p* < 0.0001). To evaluate the discriminative power of NEP to distinguish between clinically ill AKI and critically ill no-AKI patients we calculated ROC curve analysis also shown in Fig. 6 (hatched line). This resulted with an AUC of 0,68 (95% CI 0.54–0.82, SE = 0.07, *p* < 0.01) and thereby showing that NEP has little power to discriminate between these two groups.

In contrast to patients with elevated urinary NEP, 17 patients with AKI did not show NEP increase during the observation period (false negative rate of 24.64%). This might be due to the fact that the underlying pathology for AKI is not located at the proximal tubular site. These patients presented with dehydration (*n* = 2), ascending urinary tract infection (*n* = 2), cardiogenic shock (*n* = 4), multi-organ failure due to septic shock (*n* = 5) and AKI after respiratory failure (*n* = 2). There was one case of spontaneous bacterial peritonitis and one patient with AKI after chemotherapy.

In addition, 9 out of 22 patients (40.91%) in the non-AKI group showed higher NEP levels (false positive rate of 40.91%). In 4 of the 9 patients, contrast media application due to cardiac catheterization or computed tomography could be delineated shortly before NEP increase as a possible underlying cause. One patient suffered from severe gastrointestinal chemical burn with normal renal parameters. Whether these conditions indeed caused high NEP levels could not be proven within the framework of this study. Most importantly, a technical term for describing this entity has recently been established, which describes a KDIGO/RIFLE negative but biomarker positive, so called “non-creatinine-increased AKI” (NCRIAKI) [33]. Other authors refer to this entity as subclinical AKI [34].

**Fig. 7:**
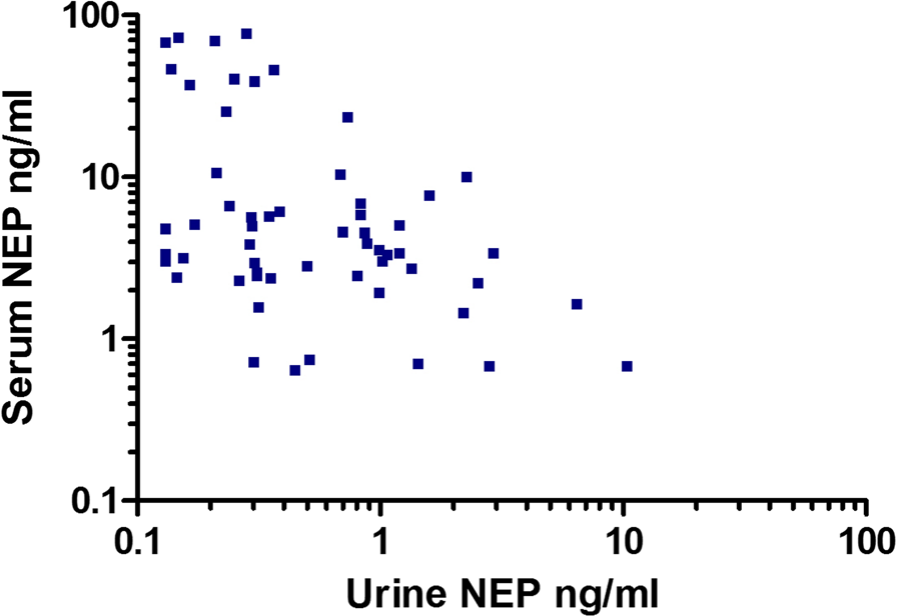
Correlation analysis of plasma and urine NEP. *Scatter plot* of correlation of plasma and urine NEP in 57 time-matched samples obtained from 21 patients

Moreover, we evaluated time-matched plasma samples (*n* = 57) obtained from 21 patients and found no correlation of plasma levels with those in urine (Pearson r − 0.2, 95% CI, −0.46-0.03). Interestingly, in 5 patients presenting with NEP of more than 25 ng/ml and up to 80 ng/ml in plasma due to pulmonary infections, the urine level was below 1 ng/ml (Fig. 7).

## Discussion

In this study we investigated urinary NEP in critically ill patients admitted to the ICU at the Medical University of Vienna.

Two major facts could be elaborated within the framework of this study; on one hand, this study investigated sequential urinary NEP levels in sepsis patients and acquired new information regarding the course of its excretion. It demonstrated high levels of NEP in AKI patients. However, no correlation with KDIGO stages of AKI and urinary NEP was found. An underlying cause might be that the extent of NEP expression at the proximal tubule varies among individuals depending on genetic markers and various premedication [35]. On the other hand, this study demonstrates that not all AKI patients (*n* = 17) present with elevated NEP. This can be explained by the fact that the pathology occurs in specific cases at sites other than the proximal tubular epithelium. The peritubular capillary endothelia represent a vulnerable cell type [36–38]. This confirms well-known facts that AKI is a complex and widely heterogeneous disorder, even among critically ill and sepsis patients. It has been demonstrated that septic AKI seems to affect the medullary thick ascending limbs and medullary collection ducts to a higher extend than proximal tubular regions [39]. In order to exclude any relation between plasma and urine NEP concentration, we elaborated plasma/urine ratios of NEP in 57 samples. There was no correlation, which was especially well documented in 4 patients who had plasma levels between 20 and 80 ng/ml, whereas their urinary NEP was below 1 ng/ml in time-matched samples.

A second important observation was that NEP levels in general were higher among ICU patients not suffering AKI according to the KDIGO criteria in comparison to healthy controls. It is conceivable that tubular cell lesions with loss of brush border proteins are common in ICU patients with the absence of overt signs of kidney dysfunction, such as those measured in terms of serum creatinine. Experts in this field attribute the delayed diagnosis in sepsis patients to reduced production of creatinine, increased distribution volume and fluid overload [40]. It is important to note that these very minor changes in serum creatinine seem to be relevant to the medical history of patients [41, 42]. Such forms of subclinical AKI have been termed “non-creatinine-increased AKI” (NCRIAKI), analogous to the non-ST elevation myocardial infarction (NSTEMI) in cardiological sub-classification of myocardial ischemia [33]. In this respect, it is noteworthy that urinary biomarkers are gaining more importance in early diagnosis of AKI at various clinical settings [43, 44]. Since AKI does not have any specific symptoms, it is even more important to improve early marker diagnostics beyond serum creatinine and urine output as these only represent late-outcome markers. Furthermore, multiple “kidney attacks” can occur in intensive care settings, which can be detected with urinary biomarkers but not using the KDIGO/RIFLE criteria. Progress in this direction can improve the outcome of AKI, which has been absent in recent years.

With the aim of explaining the high rate of recovery in our study group, we have to mention that tubular regeneration processes play a part in modulating the outcome. These regenerative processes are thought to repair such small cellular impairments [45, 46]. In this respect, during our study all patient records were looked up again in retrospect and in most patient data a potential toxic insult could be attributed to the point in time when a NEP increase occurred.

It has been clearly demonstrated in experimental models that NEP is specifically expressed at the proximal tubular cells [32]. This protein has been previously measured in patients undergoing open heart surgery and was thereby presented as an early detection marker for AKI with its peak level 3–36 h after surgery [28]. In addition, it has very recently become a promising therapeutic target in cardiovascular medicine following the development of specific inhibitors [21]. Whether this might be relevant to AKI has not been investigated.

Although NEP seems to be a good indicator of tubular alterations, we have made a striking observation that the normal urinary excretion rate of NEP is gender-dependent. Higher levels in male donors were found which is due to the fact that the prostate secrets some of this enzyme [47]. In female healthy controls the secreted NEP level is below the detection limit of this ELISA test method.

NEP represents a new urine marker protein indicative of proximal kidney tubule cell injury. A limitation of the study is that no conclusion can be drawn on the effect or clinical relevance beyond the advice to apply supportive measures to prevent progression of renal failure in patients with elevated NEP, as they are at high risk. This pilot study performed with a specific patient group will gain importance when a validation study is performed on a wider disease range and a higher patient number. Further studies are warranted in order to elaborate on whether patients undergoing NEP+ urine tests will develop chronic kidney disease during their further life span.

In recent months it has proved to be clear that a single biomarker would not suffice for the diagnosis of acute kidney injury, instead, it has been elaborated that a consortium of peptides such as KIM-1, NGAL, IL18 and IGFB7, TIMP-2 will be indicative for injuries of the nephron. In this respect, they might be more predictive when considered individually in specific clinical settings, as it has been shown with the NephroCheck – being the method of choice at the ICU or in the case of sudden deterioration of a critically ill patient [48].

## Conclusions

Elevated urinary NEP is indicative of proximal tubular cell stress or injury. As not all AKI patients show this urinary protein, various injury sites at the nephron have to be suggested depending on the type and cause of AKI. Moreover, extended tubular damage cannot be assessed by urinary NEP levels. This study confirms the previous work of others that a “non-creatinine-increased AKI” (NCRIAKI) or in other words “subclinical AKI” has to be introduced, which was used for patients suffering episodes with elevated AKI markers.

## Abbreviations

AKI: Acute kidney injury
AUC: Area under curve
BSA: Blocking solution
CKD: Chronic kidney disease
ESRD: End stage renal disease
FeNa: Fractional excretion of sodium
GFR: Glomerular filtration rate
ICU: Intensive care unit
IGFBP 7: Insulin like growth factor 7
KDIGO: Kidney disease improving global outcomes
KIM-1: Kidney injury molecule-1
NCRIAKI: Non-creatinine increase AKI
NEP: Neprilysin
NGAL: Neutrophil gelatinase-associated lipocalin
NSTEMI: Non-ST elevation myocardial infarction
PBS: Phosphate-buffered saline
PTC: Proximal tubular cells
ROC: Receiver operating characteristic
RPM: Revolution per minute
RRT: Renal replacement therapy
SBP: Spontaneous bacterial peritonitis
sCr: Serum creatinine
TIMP-2: Tissue inhibitor of metalloproteinase-2
TPBS: Tween PBS
